# Treatment outcomes and plasma level of ritonavir-boosted lopinavir monotherapy among HIV-infected patients who had NRTI and NNRTI failure

**DOI:** 10.1186/1742-6405-6-30

**Published:** 2009-12-23

**Authors:** Weerawat Manosuthi, Sasisopin Kiertiburanakul, Wannarat Amornnimit, Wisit Prasithsirikul, Supeda Thongyen, Samruay Nilkamhang, Kiat Ruxrungtham, Somnuek Sungkanuparph

**Affiliations:** 1 Bamrasnaradura Infectious Diseases Institute, Ministry of Public Health, Nonthaburi, 11000, Thailand; 2 Faculty of Medicine Ramathibodi Hospital, Mahidol University, Bangkok, Thailand; 3 The HIV Netherlands-Australia-Thailand (HIV-NAT) Research Collaboration, Thai Red Cross AIDS Research Centre, Bangkok, Thailand; 4 Department of Medicine, Faculty of Medicine, Chulalongkorn University, Bangkok, Thailand

## Abstract

**Background:**

Different strategies of ritonavir-boosted lopinavir monotherapy have been explored; however, data regarding salvage therapy among HIV-infected patients who failed nucleoside reverse transcriptase inhibitor (NRTI) and non-nucleoside reverse transcriptase inhibitor (NNRTI) is still limited.

**Methods:**

A prospective study was conducted among HIV-infected patients who failed NNRTI-based antiretroviral therapy with M184V, TAMs, and NNRTI mutations, and were naïve to protease inhibitor. LPV/r at 400/100 mg and lamivudine 150 mg were given twice daily. CD4 and HIV-1 RNA were monitored at week 0, 12, 24, and 48. LPV Cmin was assayed for the first 14 patients using HPLC.

**Results:**

There were 40 patients with a mean age of 37 years and 70% were male. Median (IQR) baseline CD4 was 123 (37-245) cells/mm^3 ^and median (IQR) HIV-1 RNA was 55,800 (9,670-100,000) copies/mL. By intend-to-treat analysis, 30 (75%) and 24 (60%) patients achieved HIV-1 RNA at <400 and <50 copies/mL, respectively. In as-treated analysis, the corresponding rates were 29 (83%) and 23 (67%), respectively. Low-level viral rebound was found in 6 (15%) patients at week 48. Medians CD4 at week 12, 24, 36 and 48 were 249, 283, 307, and 351 cells/mm^3 ^and significantly changed from baseline (all, *P *< 0.05). At 6 and 12 weeks, median (min-max) LPV Cmin was 6.52 (1.62-11.64) mg/L and 5.79 (0.75-16.31) mg/L, respectively. There were increments of mean total cholesterol and triglyceride at 48 weeks from baseline (*P *< 0.05).

**Conclusion:**

LPV/r monotherapy with recycled lamivudine can maintain virological suppression in a substantial proportion of patients failing NNRTI-based regimen and provides adequate plasma concentrations of LPV although the incidence of low-level viremia is relatively high.

## Introduction

Currently, non-nucleoside reverse transcriptase inhibitor (NNRTI)-based highly active antiretroviral therapy (HAART) is widely prescribed as an initial therapy for treatment naïve HIV-infected patients, particularly in many resource-constrained countries [1]. However, in patients who have delayed detection of treatment failure in this setting, the virus is often resistant to most existing nucleoside reverse transcriptase inhibitors (NRTIs) and NNRTIs even failing from the first regimen [2]. As a consequence, constructing the potent salvage regimens that combined 2 or 3 fully active drugs from existing drug classes is often impossible in many resource-constrained countries where new agents, such as integrase inhibitor and chemokine receptor antagonist, are neither available nor affordable. Nevertheless, the goal of attaining undetectable plasma HIV-1 RNA is remain mandatory [3]. To date, several clinical studies derived from the western countries that included 2 or more active drugs clearly demonstrate effective therapeutic strategies for antiretroviral (ARV)-experienced HIV-1 infected patients [4,5]. Hence, using ritonavir-boosted protease inhibitor in a salvage therapy was considered to be an option in the resource-constrained countries and the limitations of remaining active NRTIs usually lead to ritonavir-boosted protease inhibitor monotherapy as a salvage regimen.

Among several previous reports using ritonavir-boosted protease inhibitor, ritonavir-boosted lopinavir monotherapy has been extensively studied so far [6]. Different strategies of ritonavir-boosted lopinavir monotherapy have been explored; however, most related clinical trials studied this regimen as either a treatment simplification strategy or induction therapy in treatment-naïve patients [6]. A strategy to use ritonavir-boosted lopinavir monotherapy as a salvage regimen is not available. On the other hand, previous studies showed that continuation of lamivudine after emerging of the M184V mutation had somewhat benefit on immunological response and clinical progression in patients who had limited options of salvage regimens [7]. Moreover, there is neither additional any other mutation nor increase resistance to other antiretroviral drugs. Thus, this is the reason why we added lamivudine to decrease viral fitness in the study regimen. The objective of this study was to assess 48-week treatment responses, tolerability, and steady-state minimum plasma concentrations of ritonavir-boosted lopinavir monotherapy for salvage therapy in HIV-1 infected patients who failed antiretroviral regimens containing NRTI and NNRTI.

## Materials and methods

All patients followed at Bamrasnaradura Infectious Diseases Institute, Ministry of Public Health, Thailand, between April 2007 and February 2008 were evaluated for antiretroviral therapy failure based on guidelines for antiretroviral therapy of the Thai AIDS Society, which define failure as viral load >1,000 copies/mL after 6 months of receiving treatment or a rebound of viral load to >1,000 copies/mL in any duration after undetectable viral load [8]. Inclusion criteria were as follows: (1) HIV-1 infected patients >18 years of age, (2) failed NNRTI-based antiretroviral therapy with M184V, thymidine analogue mutations (TAMs) and NNRTI-associated mutations and (3) had plasma HIV-1 RNA >1,000 copies/mL. The patients were excluded if they had a history of exposure to protease inhibitor or receipt a medication that has drug-drug interactions with lopinavir. All patients were followed until 48 weeks of treatment. Ritonavir-boosted lopinavir in soft gel formulation at 400/100 mg and lamivudine at 150 mg were given twice daily. Clinical characteristics and findings from physical examinations were recorded for each patient. Patients were assessed as well as CD4 cell counts (flow cytometry) and plasma HIV-1 RNA (Roche Amplicor, version 1.5) at follow-up visits at weeks 0, 12, 24, and 48. The lower limit of detection for the HIV-1 RNA level is 50 copies/mL. Virological failure was defined as either a plasma HIV-1 RNA level >1,000 copies/mL after having a previously undetectable value. Serum was obtained at 24 hours after dosing to assay lopinavir concentration for the first 14 patients at the HIV Netherlands-Australia-Thailand Clinical Research Laboratory, which is located at the Chulalongkorn Medical Research Center (Bangkok), by high-performance liquid chromatography. This assay was performed in accordance with the protocol developed by the Department of Clinical Pharmacology at the University Medical Centre Nijmegen (Nijmegen, the Netherlands) [9].

All analyses were performed using SPSS, version 14.0. Mean values (± standard deviations) or median values (with interquartile ranges; IQRs) and frequency were used to describe the patients' characteristics for continuous and categorical data, respectively. The proportion of patients with plasma HIV-1 RNA <50 copies/mL after 48 weeks of ART were analyzed as intend-to-treat and as-treated. Paired t-test was used to compare parameters between time points. *P *values < 0.05 were considered to be statistically significant. The institutional ethics committees of Bamrasnaradura Infectious Diseases Institute and Ministry of Public Health approved the study. All patients signed the inform consent.

## Results

Table 1 summarizes subject characteristics and laboratory parameters. There were 40 patients with 70% male and a mean age of 37 years. The frequencies of thymidine analogue associated mutations (TAMs) were 17 (43%) D67N, 16 (40%) T215FY, 8 (20%) M41L, 6 (15%) K60R, 6 (15%) L210W, 2 (5%) K219Q. Q151M and L74V were found in 7 (18%) and 2 (5%), respectively. The prevalence of patients with ≥1 major mutation conferring drug resistance to NNRTIs was 100%.

**Table 1:** Baseline characteristics of 40 HIV-infected patients

Baseline characteristics	Number (%), n = 40
*Demographics*	

Age, mean ± SD, years	37.1 ± 8.0

Male gender, number (%)	28 (70)

Body weight, mean ± SD, Kilograms	57 ± 21

Body mass index, mean ± SD	22.7 ± 4.6

Duration of previous ART, median (IQR), years	2.1 (0.8-3.2)

*Laboratory parameters*	

CD4 cell counts at virological failure, mean ± SD, cells/mm^3^	144 ± 124

Percentage of CD4 cell at virological failure, mean ± SD, %	7.7 ± 5.1

Plasma HIV-1 RNA at virological failure, median (IQR), copies/mL	55,800 (9,670-100,000)

Log, copies/mL	4.8 (4.0-5.0)

Total cholesterol, mean ± SD, mg/dL	165 ± 42

HDL choleterol, mean ± SD, mg/dL	41 ± 30

Triglyceride, mean ± SD, mg/dL	172 ± 117

Total cholesterol:HDL ratio ≥6.5, number (%)	2 (5)

Fasting blood sugar, mean ± SD, mg%	100 ± 27

Patients with ≥3 TAMs, number (%)	13 (32.5%)

Patients with Q151M, number (%)	7 (18%)

**Figure 1:**
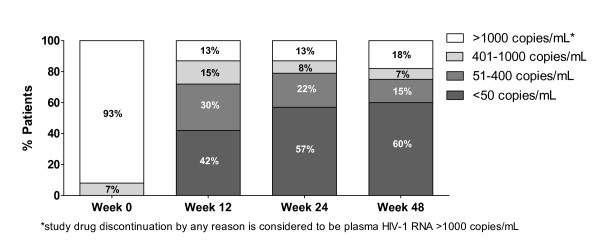
**Percentage of patients who had different stratum of plasma HIV-1 RNA at weeks 48 of treatment by intend-to-treat analysis**.

**Figure 2:**
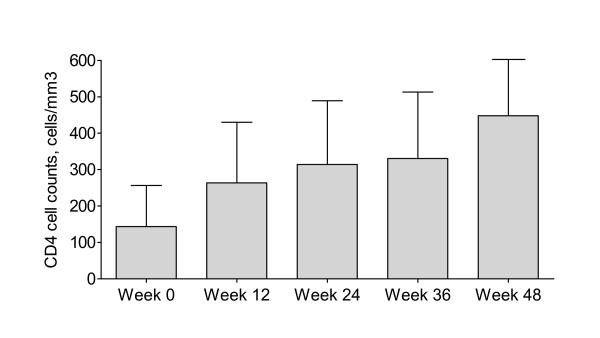
**Means and its standard deviations of CD4 cell counts changes at weeks 12, 24, 36 and 48 of treatment**.

The proportion of patients who had different stratum of plasma HIV-1 RNA at weeks 48 of treatment by intend-to-treat analysis was displayed in figure 1. By intend-to-treat analysis, 33 (83%), 30 (75%) and 24 (60%) patients achieved plasma HIV-1 RNA at <1000, <400 and <50 copies/mL after 48 weeks, respectively. In as-treated analysis (excluded dropped out, study drug discontinuation by any reason and transferred), the corresponding rates were 33 (94%), 29 (83%) and 23 (67%), respectively. Low-level viral rebound, defined as having plasma HIV-1 RNA between 50 and 400 copies/mL after having <50 copies/mL during the follow-up period, was found in 6 (15%) patients at week 48. Nine of 11 patients with plasma HIV-1 RNA >50 copies/mL had value between 50 and 1,000 copies/mL. In 11 patients, all but two had never achieved plasma HIV-1 RNA <50 copies during the study period. Two remaining patients developed virological rebound at week 48. No major PRAM was found in 2 patients who had virological rebound above 1000 copies/mL. Means CD4 cell counts changes at weeks 12, 24, 36 and 48 were shown in Figure 2. These measures significantly changed from baseline (all *P *values < 0.05). At 6 and 12 weeks of treatment, median (min-max) lopinavir minimum concentrations were 6.52 (1.62-11.64) mg/L and 5.79 (0.75-16.31) mg/L, respectively. All but one patient achieved lopinavir minimum concentrations greater than the recommended minimum concentration that was 1 mg/L. With regard to adverse reactions, 1 patient discontinued ritonavir-boosted lopinavir due to diarrhea after the first 2 weeks of ritonavir-boosted lopinavir treatment. Compared measures at week 48 to baseline values, there were increments of mean total cholesterol (206 mg/dL vs. 170 mg/dL, *P *< 0.05) and mean triglyceride (348 mg/dL vs. 216 mg/dL; *P *< 0.05). Eight patients and 24 patients had total cholesterol to HDL ratio ≥6.5 and ≥4 at week 48, respectively. Three patients needed to initiate anti-lipid agents during the follow-up period. There was an increment of fasting blood sugar from week 48 to baseline value (110 mg% vs. 100 mg%, *P *= 0.210). Two patients were receiving oral hypoglycemic agents at enrollment and another two patients had started oral hypoglycemic agents during the follow-up period.

## Discussion

As NNRTI-based HAART regimen is extensively prescribed in many resource-limited countries and due to delayed detection of virological failure from lacking the effective monitoring tools and/or inadequate infrastructure in the real-life practice, extensive NRTI and NNRTI-associated mutations could be problematic in those area. To the best of our knowledge, this is the first clinical trial that has shown clinical outcome and pharmacokinetic measures of ritonavir-boosted lopinavir together for the patients experienced with NRTIs and NNRTIs although it was conducted in a small study. Our data indicate that ritonavir-boosted lopinavir monotherapy combined with recycled lamivudine can maintain virological suppression in a substantial proportion of patients who were failing NNRTI-based regimens with M184V, TAMs and NNRTI mutations but were naïve to protease inhibitor. Using intend-to-treat analysis, 60% of patients reached the virological success, i.e., plasma HIV-1 RNA <50 copies/mL, after 48 weeks of treatment. This result is relatively consistent with a recent report that involved antiretroviral-naïve patients by Delfraissy and colleagues [10]. Of 84 patients in ritonavir-boosted lopinavir monotherapy arm, 67% and 79% had achieved undetectable plasma HIV-1 RNA by intend-to-treat and as-treated analysis, respectively. In addition, Gathe and colleagues also reported 77% of antiretroviral naïve patients who were received treatment with ritonavir-boosted lopinavir had undetectable plasma HIV-1 RNA [11]. Regarding immunological response, a higher CD4 cell count while receiving antiretroviral treatment gained, a lower risk for AIDS-related events was associated [12,13]. The present study reveals that ritonavir-boosted lopinavir showed a great performance on the immunological response after 48 weeks of treatment. A previous study showed that a regimen of ritonavir-boosted lopinavir plus 2 NNRTIs had a greater CD4 cell count response when compared with efavirenz plus 2 NRTIs [14].

Interestingly, 15% of our patients had low level viremia. This number is considered to be a significant proportion. Of this proportion, 5% achieved undetectable plasma HIV-1 RNA and 10% still had low level viremia at 72 weeks (data not shown). One possible explanation is the lack of viral suppression in some compartments, such as genital secretion and cerebrospinal fluid [15]. Another previous proposed explanation is an alternative pathway of protease inhibitor resistance facilitated by the absence of the NRTI drugs, different HIV subtypes influence on polymorphisms, and a non-adherence issue [16,17]. There was an increasing risk for virological failure in patients with suboptimal adherence. The current guidelines from Department of Health and Human Services (DHHS) suggested minimum target for lopinavir at >1 mg/L [5]. Therefore, almost all of our patients had their minimum concentration levels exceed the target cut-off value. However, a single therapeutic drug monitoring cannot exclude non-adherence, long-term follow-up is therefore warranted.

Regarding adverse reactions, the most commonly reported side effect associated with lopinavir/ritonavir is mild to moderate diarrhea [18,19]. In addition, nausea and vomiting, changes in blood lipid levels, elevated transaminase levels and altered blood glucose profiles also have been widely reported [19]. One of our patients needed to discontinue ritonavir-boosted lopinavir due to gastro-intestinal adverse event. Lopinavir/ritonavir soft gel capsules had been used in the present study instead of new lopinavir/ritonavir tablet because tablet formulation had not been available in the country during the study period. The lopinavir/ritonavir tablet formulation could lower the rate of adverse gastro-intestinal symptoms associated with the soft-gel capsules. Although this study was not designed to directly assess metabolic complication, it could revealed a great impact of ritonavir-boosted lopinavir on serum lipid parameters, included total cholesterol, LDL-cholesterol, triglyceride and total cholesterol to HDL-cholesterol ratio, at 48 weeks of treatment. Likewise, a proportion of patients had total cholesterol to HDL-cholesterol ratio above cut-off value; i.e. ≥4, that related to high risk of coronary heart disease [20,21].

In treatment-experienced patients failing NNRTI-based regimens with limited NRTI options, switching to at least 2 fully active drugs to an optimized antiretroviral regimen is principally the best strategy so far. In patients with earlier first-line treatment failure and have developed either only M184V or with few TAMs, the option of NRTI backbone is still not limited. In these cases, there is a possibility to include 1 or 2 active NRTI drugs in combination with a new drug class, such as PIs, to assure the effectiveness of the regimen. Thus in settings where other new ARV classes beside PIs cannot be assessable, early detect virological failure is crucial to preserve the NRTI backbone. While waiting for a randomized control trial to prove HIV monotherapy with ritonavir-boosted protease inhibitor in these particular patients, the present data provide support to physicians currently facing choices of salvage regimen options in many resource-constrained countries. Ritonavir-boosted lopinavir combined with recycled lamivudine to decrease viral fitness can maintain virological suppression in a substantial proportion of patients failing NRTI and NNRTI with M184V and provides adequate plasma concentrations of lopinavir although incidence of low-level viremia is relatively high. A further larger study would be required to assess the risk and benefit of this proposed strategic treatment in the resource-constrained settings.

## Competing interests

The authors declare that they have no competing interests.

## Authors' contributions

WM participated in the design of the study, statistical analysis and draft the manuscript. SK, WA, WP, ST, KR and SS participated in the design of the study and draft the manuscript. SN participated in the design of the study. All authors read and approved the final manuscript.

## Funding Statement

This study was supported by research grants from Bamrasnaradura Infectious Diseases Institute, Thailand.
